# Therapeutic effect of vasoactive intestinal peptide on form-deprived amblyopic kittens

**DOI:** 10.1186/s12886-019-1203-1

**Published:** 2019-08-20

**Authors:** Bo Li, Yunchun Zou, Liwen Li, Hongwei Deng, Wei Mi, Xing Wang, Ximin Yin

**Affiliations:** 1 Department of Optometry, North Sichuan Medical College, Nanchong, 637000 Sichuan People’s Republic of China; 2 Department of Ophthalmology, Suining Central Hospital, Suining, 629000 Sichuan People’s Republic of China

**Keywords:** Amblyopia, Kitten, Lateral geniculate body, Vasoactive intestinal peptide

## Abstract

**Background:**

Exploring the role of vasoactive intestinal peptide (VIP) in the lateral geniculate body (LGBd) in visual development and studying the therapeutic effect of VIP on amblyopic kittens.

**Methods:**

Three-week-old domestic cats were divided into a control group (*n =* 10) and a monocular deprivation group (*n* = 20), with an eye mask covering the right eye of those in the deprived group. After pattern visual evoked potential (PVEP) recording confirmed the formation of monocular amblyopia, the left LGBd was isolated from 5 kittens in each group. The remaining control kittens continued to be raised, and the remaining deprivation group was divided into a VIP intervention group (*n* = 5), Sefsol (caprylic acid monoglyceride, VIP solution) intervention group (*n =* 5) and amblyopia non-intervention group (*n* = 5) after removal of the eye mask. Three weeks later, PVEPs, VIP immunohistochemistry and VIP mRNA expression in the left LGBd were compared across groups.

**Results:**

At 6 weeks of age, there were significant differences in P100 wave latency and amplitude and VIP immunohistochemistry and in situ hybridization between the control group and the deprivation group (*P* < 0.05). After 3 weeks of the corresponding interventions, the latency and amplitude in the VIP intervention group were better than that in the Sefsol intervention group and amblyopia non-intervention group (*P* < 0.05). Furthermore, VIP treatment increased the number of immunohistochemical VIP-positive cells (*P* < 0.05) and the average optical density of positive cells (*P* > 0.05), as well as the number (*P* < 0.05) and average optical density of VIP mRNA-positive cells (*P* < 0.05).

**Conclusions:**

VIP plays an important role in visual development. Nasal administration of VIP can improve the function of neurons in the LGBd of kittens and has a certain therapeutic effect on amblyopia.

## Background

Molecular biology has allowed for a deeper investigation of changes in the visual nervous system associated with amblyopia [1]. The lateral geniculate body (LGBd) is part of the visual nervous system that participates in the formation of fine vision, such as directionality [2]. In amblyopia, neuronal function in the LGBd is reduced [3] or even atrophied [4, 5]. The afferent links in areas 17 and 18 of the cat’s visual cortex are formed by neurons in the dorsolateral geniculate body [6], and the lateral geniculate neurons projecting to area 17 of the visual cortex remain plastic in adult mice [7]. So the LGBd plays an important role in visual development. Scholars have proven that many neurotransmitters, such as nerve growth factor, brain-derived neurotrophic factor, they are beneficial for the transmission of nerve signals or for the nutrition of neurons, and the expression of these neurotransmitters is significantly inhibited during amblyopia. Therefore, studying how a specific neurotransmitter is altered in the amblyopic LGBd can reveal the role of this transmitter in visual development, and the potential therapeutic effect of this neurotransmitter on amblyopia can be observed in amblyopic animals, thereby providing a possible theoretical basis for the treatment of amblyopia.

As a neurotransmitter, vasoactive intestinal peptide (VIP), is composed of 28 amino acid residues and belongs to the secretory glucagon family. VIP was named for its vasodilation activity and was initially considered a candidate gastrointestinal hormone. VIP has been found to be widely distributed in the cerebral cortex and intraocular tissue [8–11]. The cerebral cortex of rats began to express VIP at birth, and VIP expression in the cortex was significantly up-regulated 42 days ago, and the expression was significantly down-regulated from 42 days to adulthood [12]. As a neuromodulator, VIP can inhibit the development of form-deprivation myopia [13]. Subsequently, VIP was shown to be widespread in the LGBd [14].

The purpose of this study was to explore the role of VIP in the LGBd of kittens in visual development. At the same time, our study aimed to provide a relatively safe and effective drug treatment for amblyopia that could have a high utilization rate and be electrophysiologically effective.

## Methods

### Animals

We used thirty healthy 3-week-old domestic cats (The Experimental Animal Centre of North Sichuan Medical College, Nanchong, China. Production approval number: SCXK (Liao) 2018–0003. Application approval number: SYXK (Chuan) 2019–215), regardless of gender and hair colour, weighing approximately 290 g — 360 g and excluded those with refractive medium opacity and fundus abnormalities. Optometry revealed that the dioptre of all kittens was about + 1.0 D − + 2.5 D. The kittens were kept in an environment (about 50mm^2^) with sufficient light, no social isolation and, and an indoor temperature maintained at (27 ± 1) °C, provided with enough toys, cat scratching boards and cat litter in the room (provided by the Experimental Animal Centre of North Sichuan Medical College). Before 5 weeks of age, kittens are unable to eat solid food autonomously; therefore, the kittens were fed kitten milk powder and drinking water regularly 8 times a day. After 5 weeks of age, the cats had access to sufficient amounts of fresh food and drinking water indoors. This study was approved and supervised by the Experimental Animal Ethics Committee of North Sichuan Medical College.

### Animal model establishment

**Fig. 1:**
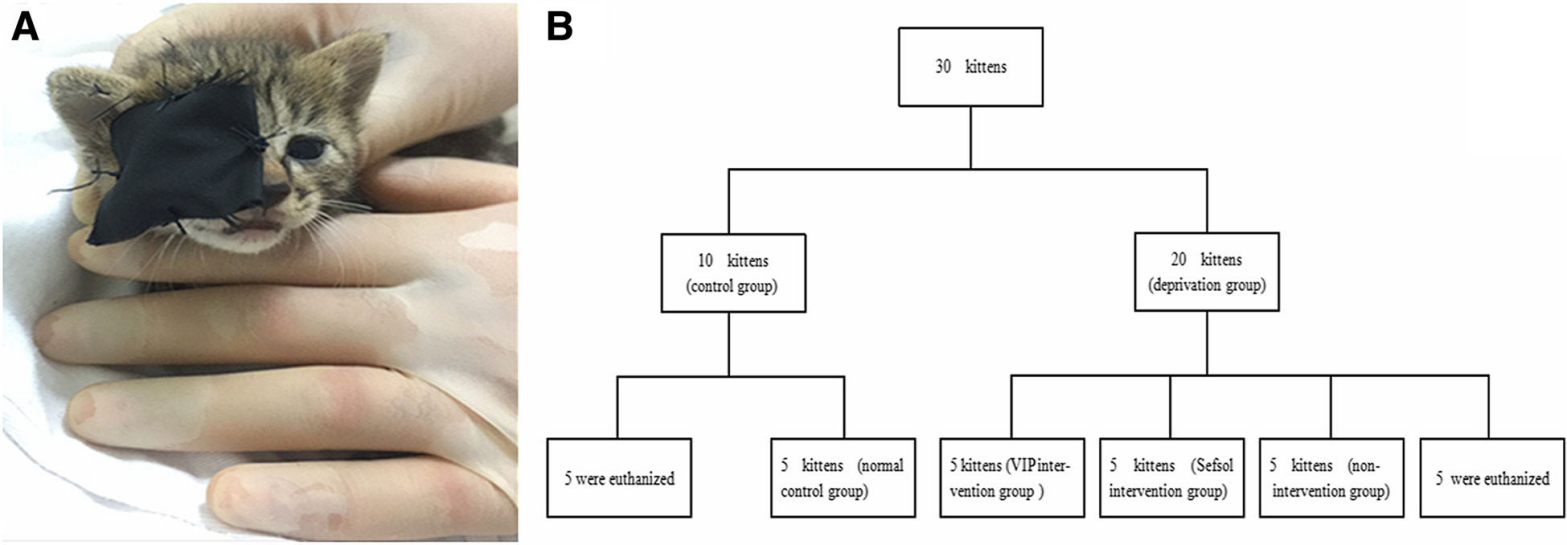
Cover model and grouping diagram of kittens. **a** The right eyes of the deprivation group were covered with an eye. Patch; **b** Schematic diagram of the grouping of kittens during the study

The kittens were randomly divided into a control group (*n =* 10) and a monocular deprivation group (*n =* 20) (randomly designated by non-breeders). The kittens in the deprivation group were anesthetized by injecting intraperitoneally with 2% sodium pentobarbital (provided by the Experimental Animal Centre of North Sichuan Medical College, Nanchong, China) (35 mg/kg) in the operating room, paying close attention to breathing during anesthesia, using a table lamp to maintain body temperature. After confirmation that the kitten had no reaction to the pain in the ear, four skin fixation sutures were made symmetrically in the right orbital skin. The end of the suture was formed into a small ring (that always existed during the covering process), through which a silk thread was passed. Then, a knot was tied through four holes of a black blindfold to fix the blindfold and ensure that it does not oppress the eyeball (Fig. 1a). After disinfecting the skin suture with iodophor, the kittens were wrapped with the appropriate size of quilt and pay attention to their status. At the age of 6 weeks, the PVEP recordings were compared between groups to ensure that the deprivation group had acquired monocular amblyopia. Five kittens in each group were randomly selected, and each kitten was euthanized by injecting intraperitoneally with 2% sodium pentobarbital (100 mg/kg). The left LGBd was isolated according to the Sinder cat brain stereotactic map. The remaining 5 kittens in the control group continued to be raised as the normal control group. After the blindfold was removed, the remaining 15 amblyopia kittens were randomly divided into a VIP intervention group, which received 10 μg VIP/40 μl (containing 10% Sefsol and 40% isopropanol) daily through nasal mucosa; a Sefsol intervention group, which received 10% Sefsol and 40% isopropanol via nasal mucosa every day; and a amblyopia non-intervention group, which did not receive any treatment (Fig. 1b). All kittens were euthanized by 2% sodium pentobarbital (100 mg/kg) at 9 weeks of age. VIP immunohistochemistry and VIP mRNA in situ hybridization were then performed on all samples. Throughout the study, no kittens died unexpectedly.

### PVEP detection

PVEP was tested every other week after the models were established. After intraperitoneal injection of 2% sodium pentobarbital (35 mg/kg), the hair of the corresponding part of the kitten was removed. Three needle electrodes were sterilized by alcohol and used to penetrate the skin of forehead, occipital and the back of the ear. The corresponding lens was used to correct the refraction, and the head position was adjusted so that the centre of the posterior pole of the retina was on the same horizontal line as the centre of the screen. PVEP was detected with checkerboard reversal stimulation with a mode of 0.3 cpd, time frequency of 1 Hz, and 64 superpositions. PVEP detection was repeated on each eye three times.

### VIP immunohistochemistry

Paraffin sections were dewaxed to water and placed in a repair box containing citric acid (PH6.0) antigen repair buffer for antigen repair. The slices were placed in 3% hydrogen peroxide solution and phosphate buffer saline (pH = 7.4) in turn to block endogenous peroxidase. The tissue was then evenly covered with 3% BSA blocking solution (Seville Co., Ltd. Wuhan, China) in the culture dish for serum blocking. The VIP antibody and HRP-labelled goat anti-rabbit antibody were added in sequence, and the tissue was developed with diaminobenzidine. Positive staining appeared as a brownish yellow colour. Haematoxylin was used to dye the nucleus blue, and the tissue was dehydrated. Microscopic examination, image acquisition and analysis were then performed.

### VIP mRNA in situ hybridization

Paraffin sections were dewaxed in water and boiled in repair solution for 10 min. After natural cooling, digested with protease K (20 μg/ml) at 37 °C for 25 min. Then, 3% methanol-hydrogen peroxide was added, and the slide was placed in phosphate buffer saline (PH7.4) to block endogenous peroxidase. After pre-hybridization, VIP1 + VIP2 + VIP3 mRNA probe (5′-DIG-CGAAG GCGGG TATAG TTGTC GGTGA AGA-DIG-3′;5′-DIG-TGCAT CCGAG TGGCG CTTGA TTGG-DIG-3′; 5′-DIG-CTGGT TTCCA TCTTT GTACC TTGCC AAGTA GTG-DIG-3’)hybridization solution containing the probe was added (Seville co., Ltd. Wuhan, China) at a concentration of 3 ng/μl. Hybridization was conducted at 37 °C in an incubator overnight, and then the hybridization solution was washed away. BSA blocking solution was then added, followed by a drop of mouse anti-digoxigenin-labelled peroxidase (Jackson Inc., USA). Positive VIP expression was visualized with the brownish yellow staining of diaminobenzidine, and haematoxylin stained the nuclei blue. The tissue was then dehydrated, and microscopic examination, image acquisition and analysis were performed.

### Statistical analysis

The statistical software SPSS 22.0 was used. The data were expressed as the mean ± standard deviation (^−^x ± s). The results of P100 wave, immunohistochemistry and in situ hybridization between each group were compared by Two independent sample t tests, and the rank sum test was used for data that did not obey the normal distribution.

## Results

### PVEP

**Fig. 2:**
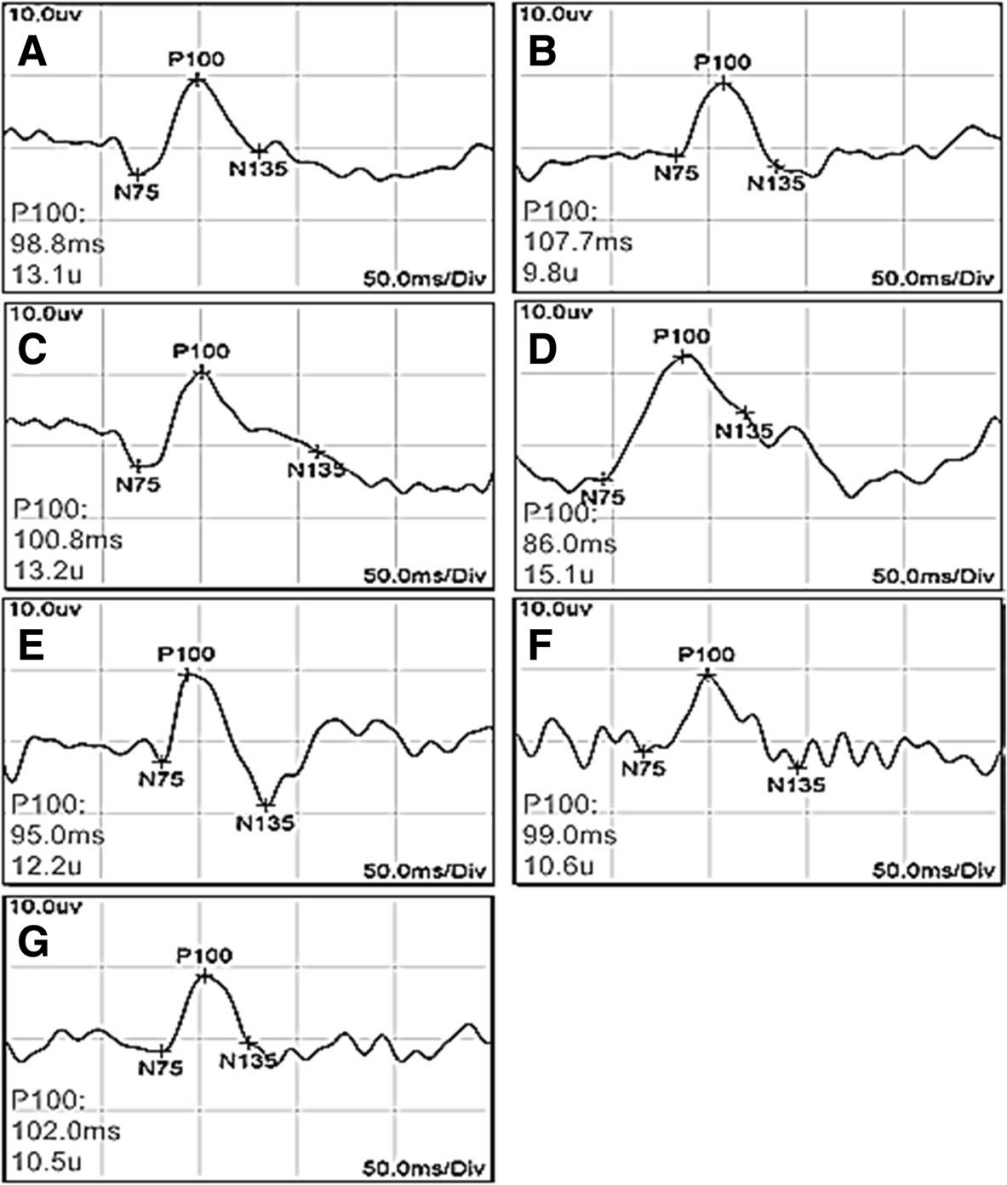
PVEP curves of each group of kittens. Figure legend: At 6 weeks of age, P100 in the (**b**) right eye of the deprivation group was compared with the (**c**) contralateral eye and the (**a**) right eye of the control group, the latency was prolonged and the amplitude decreased. At the age of 9 weeks, P100 of (**e**) VIP intervention group was compared with that of (**f**) Sefsol intervention group and (**g**) amblyopia non-intervention group, the latency was shortened and the amplitude was increased. The latency of P100 in VIP intervention group was still longer than that in (**d**) normal control group, and the amplitude was still lower

When performing a PVEP test, a good waveform can be seen by controlling the resistance (Fig. 2). At the age of 6 weeks, the latency of the right eye in the control group and the left eye in the deprivation group was shorter than right eye in the deprivation group (*P =* 0.000, 0.000), and the amplitude was higher (*P =* 0.009, 0.000), indicating the formation of monocular amblyopia in the deprivation group. At the age of 9 weeks, no obvious changes in the habits of kittens were observed, and the feeding process was normal. The latency of the P100 wave in the right eye of the VIP intervention group was shorter than that of the Sefsol intervention group (*P =* 0.015) and amblyopia non-intervention group (*P =* 0.005) but longer than that of the normal control group *(P =* 0.000). The amplitude of the P100 wave in the right eye of the VIP intervention group was higher than that of the Sefsol intervention group (*P =* 0.017) and the amblyopia non-intervention group (*P =* 0.010) but lower than that of the normal control group (*P =* 0.001). The latency and amplitude of the Sefsol intervention group was compared with those of the amblyopia non-intervention group (*P* = 0.180,0.646). Therefore, VIP had a positive effect on the recovery of visual function in amblyopic kittens (Table 1). (Relevant data is available at https://figshare.com/s/8c33cde2c66fc92d39ea).

**Table 1:** P100 latency and amplitude in each group

	P100 latency	P100 amplitude
Right eye of the 6-week-old control group	98.19 ± 1.65 ms	12.57 ± 0.93u
Right eye of 6-week-old deprivation group	110.38 ± 2.04 ms	9.62 ± 0.70u
Left eye of 6-week-old deprivation group	99.35 ± 2.04 ms	12.12 ± 0.89u
9-week-old normal control group	87.93 ± 1.71 ms	13.50 ± 0.64u
9-week-old VIP intervention group	96.23 ± 1.61 ms	11.59 ± 0.55u
9-week-old Sefsol intervention group	99.14 ± 1.34 ms	10.58 ± 0.53u
9-week-old non-intervention group	100.76 ± 2.06 ms	10.41 ± 0.57u

At 6 weeks of age, the right eye in the deprivation group had a longer latency and lower amplitude than the left eye in the deprivation group (*P* = 0.000,0.000) and the right eye in the control group (*P* = 0.000,0.000). At the age of 9 weeks, the VIP intervention group had a shorter latency and a higher amplitude than that of the Sefsol intervention group (*P* = 0.015,0.017) and the amblyopia non-intervention group (*P* = 0.005,0.010), but the latency was longer and the amplitude was lower than the normal control group (*P* = 0.000,0.001)

### VIP immunohistochemistry

**Fig. 3:**
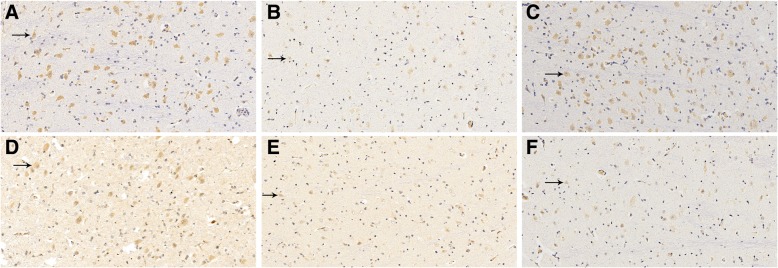
Immunohistochemical performance in lateral geniculate neurons in each group (DAB× 200). VIP positive expression in the cytoplasm of neurons was brown-yellow. At the age of 6 weeks, **a** control group had more positive cells, in the (**b**) deprivation group, there were fewer positive cells. At 9 weeks of age, **c** normal control group with more positive cells, in the (**d**) VIP intervention group, positive cells increased compared with the (**e**) Sefsol intervention group and the (**f**) non-intervention group, but still less than the normal control group

Two visual fields were randomly selected from each slice for statistical analysis. Positive VIP expression, indicated by brown to yellowish yellow staining, was found in the cytoplasm of LGBd neurons in each group, and the nuclei were stained blue (Fig. 3). At the age of 6 weeks, there were more VIP-positive cells in the control group than in the deprivation group (*P =* 0.000), and the average optical density of the positive cells in the control group was higher than that in the deprivation group (*P =* 0.000). At 9 weeks of age, the VIP intervention group had more VIP-positive cells than the Sefsol intervention group (*P =* 0.008) and the amblyopia non-intervention group (*P =* 0.017) but fewer than the normal group (*P =* 0.000). The average optical density of VIP-positive cells in the VIP intervention group was higher than that in the Sefsol intervention group (*P =* 0.015) and amblyopia non-intervention group (*P =* 0.055), but weaker than that in the normal group (*P =* 0.000). The Sefsol intervention group was compared with the amblyopia non-intervention group *(P =* 0.613,0.780). (Table 2). (Relevant data is available at https://figshare.com/s/8c33cde2c66fc92d39ea).

**Table 2:** VIP immunohistochemical results in each group

Cells	Positive cell number	Mean optical density of positive
6-week-old control group	107.00 ± 15.04	3628.46 ± 992.79
6-week-old deprivation group	38.70 ± 24.39	1836.87 ± 980.49
9-week-old normal control group	128.75 ± 15.51	4181.37 ± 811.04
9-week-old VIP intervention group	69.20 ± 11.93	2955.82 ± 816.14
9-week-old Sefsol intervention group	58.15 ± 12.93	2565.91 ± 658.85
9-week-old amblyopia non-intervention group	60.10 ± 11.20	2510.35 ± 588.32

At the age of 6 weeks, the number of VIP positive cells and the average optical density in the control group were higher than those in the deprivation group (*P* = 0.000,0.000).At the age of 9 weeks, the number of positive cells and average optical density in the VIP intervention group were higher than those in the Sefsol intervention group (*P* = 0.008,0.105) and the amblyopia non-intervention group (*P* = 0.017,0.055), but still lower than those in the normal control group (*P* = 0.000,0.000)

### VIP mRNA in situ hybridization

**Fig. 4:**
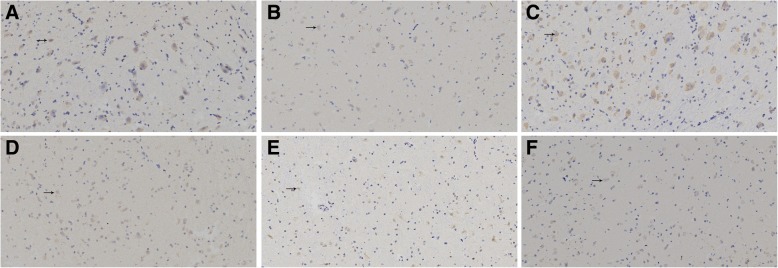
In situ hybridization performance in lateral geniculate neurons in each group (DAB× 200). The positive expression of VIP-mRNA was found in the cytoplasm of the neurons and overlapped with the blue staining nuclei, showing a brown-yellow color. At the age of 6 weeks, **a** control group had more positive cells, in the (**b**) deprivation group, there were fewer positive cells. At 9 weeks of age, **c** normal control group with more positive cells, in the **d** VIP intervention group, positive cells increased compared with the (**e**) sefsol intervention group and the (**f**) non-intervention group, but still less than the normal control group

Two visual fields were randomly selected from each slice for statistical analysis. VIP mRNA, indicated by brown to yellowish yellow staining, was expressed in the cytoplasm of LGBd neurons of all kittens and overlapped with the blue-stained nucleus (Fig. 4). At the age of 6 weeks, the kittens in the control group had more VIP mRNA-positive cells than those in the deprivation group (*P =* 0.000), and the average optical density of the positive cells in the control group was higher than that in the deprivation group (*P =* 0.000). At 9 weeks of age, kittens in the VIP intervention group had more positive cells than those in the Sefsol intervention group (*P* = 0.012) and amblyopia non-intervention group (*P =* 0.023) but fewer than the kittens in the normal control group (*P* = 0.000). The average optical density of positive cells in the VIP intervention group was higher than that in the Sefsol intervention group (*P* = 0.037) and amblyopia non-intervention group (*P* = 0.007) but lower than that in the normal control group (*P* = 0.000) (Table 3). (Relevant data is available at https://figshare.com/s/8c33cde2c66fc92d39ea).

**Table 3:** VIP mRNA in situ hybridization results in each group

Cells	Positive cell number	Mean optical density of positive
6-week-old control group	58.40 ± 11.32	2990.73 ± 712.81
6-week-old deprivation group	29.60 ± 9.13	1198.70 ± 305.75
9-week-old normal control group	73.25 ± 10.88	3518.02 ± 512.59
9-week-old VIP intervention group	49.35 ± 9.91	1973.34 ± 593.97
9-week-old Sefsol intervention group	41.40 ± 9.06	1624.99 ± 408.61
9-week-old amblyopia non-intervention group	42.40 ± 8.60	1522.86 ± 351.60

At the age of 6 weeks, the number of VIP positive cells and the average optical density in the control group were higher than those in the deprivation group (*P* = 0.000,0.000).At the age of 9 weeks, the number of positive cells and average optical density in the VIP intervention group were higher than those in the Sefsol intervention group (*P* = 0.012,0.037) and the amblyopia non-intervention group (*P* = 0.023,0.007), but still lower than those in the normal control group (*P* = 0.000,0.000)

## Discussion

Short-term monocular deprivation weakens the synaptic connections of the visual conduction pathway in the deprived eye, resulting in a significant decline in vision [15]. Form deprivation during the sensitive period of visual development leads to amblyopia. Our experiment was carried out during the sensitive period of visual development in kittens to ensure that this effect of form deprivation was observed. During the sensitive period of visual development, amblyopia and a decrease in VIP expression in the LGBd were caused by the unequal input of binocular visual information, and this decrease in VIP expression promoted the development of amblyopia. After 3 weeks of nasal administration of VIP, the PVEP results showed improvements, and VIP expression in the LGBd was increased. These results suggest that nasally administered VIP can reach the cerebral cortex and have an effect. VIP intervention can improve the metabolism of lateral geniculate neurons and enhance visual function during the sensitive period of visual development in amblyopic kittens.

After monocular deprivation, the expression of VIP in the LGBd of the deprivation group was significantly lower than that of the control group, indicating that VIP expression depended on normal illumination. Immunohistochemistry showed that the ability of LGBd neurons to express the VIP protein was decreased by form deprivation, and in situ hybridization demonstrated that form deprivation also affected the production of endogenous VIP mRNA in the LGBd. VIP, as a neurotransmitter in the central system [16, 17], is widely distributed in neurons [18] and binds to receptors VPAC1, VPAC2, and PAC1 [19]. After binding to the receptor, VIP plays a physiological role through a series of signal transduction pathways, including the cAMP-dependent protein kinase pathway, alcohol phospholipid pathway, ornithine decarboxylase polyamine pathway and Ras pathway. Down-regulated expression of VIP in the LGBd will lead to a reduction in the binding of VIP to its associated receptors, thereby probably inhibiting the expression of the corresponding function. At the same time, the decrease in VIP reduces the diurnal discharge frequency of neurons, thereby affecting the long-term electrical activity in the suprachiasmatic nucleus of the central system [20, 21]. The down-regulation in VIP also inhibits electrical transmission between neurons via the VPAC2-mediated cAMP pathway [22], thereby hindering the information transmission in the visual nervous system. These effects, in turn, accelerate the emergence of amblyopia.

After 3 weeks of VIP intervention, the expression of VIP in lateral geniculate neurons increased, and increased VIP expression promoted the recovery of visual function through its physiological effects. VIP can counteract the decrease in neurons caused by electrical conduction block [23], inhibit the apoptosis of neurons by reducing the translocation of cytochrome C from the mitochondria to the cytoplasm [24], and promote the proliferation of neurons. VIP can inhibit the production of interleukin-1 β, tumour necrosis factor α, β-amyloplast and other inflammatory and neurotoxic factors produced by microglia in the inflammatory environment and plays a protective role in neurons [25, 26]. VIP provides indirect nutritional support to neurons by acting on astrocytes [27] and provides nutrition for neurons undergoing division [28]. VIP has also been found to induce glycogen decomposition in the cerebral cortex of mice, potentially through increasing glucose utilization by promoting the formation of AMP [29]. VIP is also involved in regulating the secretion of intracellular pancreatic polypeptide, adiponectin, insulin and other metabolic hormones, thereby affecting cell metabolism [30]. VIP can also activate other excitatory intermediate neurons to produce excitatory postsynaptic potentials, which further increase the excitability of the central nervous system. VIP affects the biological metabolism of neurons in the visual nervous system by regulating the proliferation and differentiation of neurons, the synthesis of a variety of cytokines, the secretion of related hormones and the nutritional support of neurons to improve visual function.

Arden et al. [31] designed the checkerboard square reversal stimulation visual evoked potential test for clinical amblyopia examination. The latency of the P100 wave in amblyopia eyes is longer and the amplitude is lower than in normal eyes. Visual evoked potentials have been widely used as a diagnostic and therapeutic evaluation of amblyopia [32, 33]. In our experiment, the latency of the P100 wave in amblyopic eyes of amblyopic kittens was longer and the amplitude was lower than those in the contralateral eyes and the ipsilateral eyes of the control group, consistent with the results of previous studies. Hubel and Wiesel [34] found that the visual plasticity of kittens was highly sensitive before 8 weeks after birth, gradually decreased after 8 weeks, and disappeared at the 3rd month. After 3 months, the effect of monocular deprivation on the size of neurons in the LGBd was basically negligible [35]. Therefore, we performed monocular deprivation at the 3rd week after birth to ensure its effect on visual development. Because dark environments affect the plasticity of the visual cortex of kittens, increasing the amplitude of P100 waves [36] and improving vision [37], dark environments may also affect the plasticity of the LGBd. All experimental animals were maintained in a 24-h light environment, and observation revealed that maintaining light at night did not affect sleep. Correction of ametropia was performed during PVEP to avoid interference with P100 latency and amplitude [38]. Gozes et al. [39] administered VIP to the nasal mucosa of rats through inhalation to treat Alzheimer’s disease and found that the concentration of VIP in the brains of rats was similar to that found with direct intraventricular injection. In contrast, intravenous administration of VIP results in significantly lower concentrations in the brain and blood [40]. Therefore, VIP (containing 10% Sefsol and 40% isopropanol) was given through nasal mucosa, in which Sefsol and isopropanol were used as penetration enhancers. The latency and amplitude of the P100 wave in the amblyopia Sefsol intervention group were similar to those in the amblyopia non-intervention group, with differences that were not statistically significant; therefore, the effects of Sefsol and isopropanol as penetration enhancers on the experimental results were excluded. At the same time, innovative covering methods were used to reduce the risk of skin infection due to traditional eyelid suture and avoid the adverse consequences of repeated PVEP detection on eyelid skin, such as corneal irritation and suture rupture.

However, due to the short intervention time of VIP in this experiment, the full pharmacological effect of VIP may not have been observed; the P100 latency and amplitude in the VIP intervention group remained significantly different from those in the normal control group. Moreover, there was no significant difference in the average optical density of VIP immunohistochemically positive cells between the amblyopia Sefsol intervention group and the amblyopia non-intervention group. However, significant differences were still seen in the number of VIP-positive cells. Based on the experimental results, VIP undeniably has a therapeutic effect on the visual nervous system. Some studies have found that LGBd function in form-deprived amblyopic kittens can be partially restored after the sensitive period of visual development [41], which is worthy of our next study.

## Conclusions

In summary, VIP in the LGBd of kittens plays an important role in visual development. Nasal administration of VIP in amblyopic kittens promoted the development and growth of neurons in the LGBd, increased the expression of VIP, shortened the latency of the P100 wave and increased its amplitude in amblyopic eyes. Therefore, VIP has therapeutic significance for form-deprived amblyopic kittens.

## Data Availability

The datasets used and analysed during the current study are available from the corresponding author on reasonable request. Or all relevant datasets related to the study can be found in the specified database (https://figshare.com/s/8c33cde2c66fc92d39ea).

## Abbreviations

LGBd: lateral geniculate body
PVEP: pattern visual evoked potential
VIP: Vasoactive intestinal peptide

## References

[1] Von Noorden GK (1973). Histological studies of the visual system in monkeys with experimental amblyopia. Investig Ophthalmol.

[2] Piscopo DM, El-Danaf RN, Huberman AD, Niell CM (2013). Diverse visual features encoded in mouse lateral geniculate nucleus. J Neurosci.

[3] Wiesel TN, Hubel DH (1963). Effects of visual deprivation on morphology and physiology of cells in the cat's lateral geniculate body. J Neurophysiol.

[4] Guillery RW, Stelzner DJ (1970). The differential effects of unilateral lid closure upon the monocular and binocular segments of the dorsal lateral geniculate nucleus in the cat. J Comp Neurol.

[5] Takahata T, Patel NB, Balaram P, Chino YM, Kaas JH (2018). Long-term histological changes in the macaque primary visual cortex and the lateral geniculate nucleus after monocular deprivation produced by early restricted retinal lesions and diffuser induced form deprivation. J Comp Neurol.

[6] Toporova SN, Alekseenko SV, Makarov FN (2004). Afferent connections of fields 17 and 18 of the cat cerebral cortex formed by neurons of the dorsal lateral geniculate body. Neurosci Behav Physiol.

[7] Jaepel J, Hubener M, Bonhoeffer T, Rose T (2017). Lateral geniculate neurons projecting to primary visual cortex show ocular dominance plasticity in adult mice. Nat Neurosci.

[8] Said SI, Rosenberg RN (1976). Vasoactive intestinal polypeptide: abundant immunoreactivity in neural cell lines and normal nervous tissue. Science.

[9] Fuxe K, Hökfelt T, Said SI, Mutt V (1977). Vasoactive intestinal polypeptide and the nervous system: immunohistochemical evidence for localization in central and peripheral neurons, particularly intracortical neurons of the cerebral cortex. Neurosci Lett.

[10] Lorén I, Emson PC, Fahrenkrug J, Björklund A, Alumets J, Håkanson R (1979). Distribution of vasoactive intestinal polypeptide in the rat and mouse brain. Neuroscience.

[11] Uddman R, Alumets J, Ehinger B, Hakanson R, Loren I, Sundler F (1980). Vasoactive intestinal peptide nerves in ocular and orbital structures of the cat. Invest Ophthalmol Vis Sci.

[12] Emson PC, Gilbert RF, Loren I, Fahrenkrug J, Sundler F, Schaffalitzky de Muckadell OB (1979). Development of vasoactive intestinal polypeptide (VIP) containing neurones in the rat brain. Brain Res.

[13] Cakmak AI, Basmak H, Gursoy H, Ozkurt M, Yildirim N, Erkasap N (2017). Vasoactive intestinal peptide, a promising agent for myopia?. Int J Ophthalmol.

[14] Ogawa-Meguro R, Itoh K, Mizuno N (1992). Substance P-, vasoactive intestinal polypeptide-, and cholecystokinin-like immunoreactive fiber projections from the superior colliculus to the dorsal lateral geniculate nucleus in the rat. Exp Brain Res.

[15] Rittenhouse CD, Shouval HZ, Paradiso MA, Bear MF (1999). Monocular deprivation induces homosynaptic long-term depression in visual cortex. Nature.

[16] Bryant MG, Polak MM, Modlin I, Bloom SR, Albuquerque RH, Pearse AG (1976). Possible dual role for vasoactive intestinal peptide as gastrointestinal hormone and neurotransmitter substance. Lancet.

[17] Quik M, Iversen LL, Bloom SR (1978). Effect of vasoactive intestinal peptide (VIP) and other peptides on cAMP accumulation in rat brain. Biochem Pharmacol.

[18] Ishihara T, Shigemoto R, Mori K, Takahashi K, Nagata S (1992). Functional expression and tissue distribution of a novel receptor for vasoactive intestinal polypeptide. Neuron.

[19] Dejda A, Sokolowska P, Nowak JZ (2005). Neuroprotective potential of three neuropeptides PACAP. VIP and PHI Pharmacol Rep.

[20] Vosko A, van Diepen HC, Kuljis D, Chiu AM, Heyer D, Terra H (2015). Role of vasoactive intestinal peptide in the light input to the circadian system. Eur J Neurosci.

[21] Hermanstyne TO, Simms CL, Carrasquillo Y, Herzog ED, Nerbonne JM (2016). Distinct firing properties of vasoactive intestinal peptide-expressing neurons in the suprachiasmatic nucleus. J Biol Rhythm.

[22] Kudo T, Tahara Y, Gamble KL, McMahon DG, Block GD, Colwell CS (2013). Vasoactive intestinal peptide produces long-lasting changes in neural activity in the suprachiasmatic nucleus. J Neurophysiol.

[23] Brenneman DE, Eiden LE (1986). Vasoactive intestinal peptide and electrical activity influence neuronal survival. Proc Natl Acad Sci U S A.

[24] Antonawich FJ, Said SI (2002). Vasoactive intestinal peptide attenuates cytochrome c translocation, and apoptosis, in rat hippocampal stem cells. Neurosci Lett.

[25] Delgado M, Ganea D (2003). Vasoactive intestinal peptide prevents activated microglia-induced neurodegeneration under inflammatory conditions: potential therapeutic role in brain trauma. FASEB J.

[26] Tan YV, Waschek JA, Targeting VIP (2011). PACAP receptor signalling: new therapeutic strategies in multiple sclerosis. ASN Neuro.

[27] Brenneman DE, Neale EA, Foster GA, d'Autremont SW, Westbrook GL (1987). Nonneuronal cells mediate neurotrophic action of vasoactive intestinal peptide. J Cell Biol.

[28] Pincus DW, DiCicco-Bloom E, Black IB (1994). Trophic mechanisms regulate mitotic neuronal precursors: role of vasoactive intestinal peptide (VIP). Brain Res.

[29] Magistretti PJ, Morrison JH, Shoemaker WJ, Sapin V, Bloom FE (1981). Vasoactive intestinal polypeptide induces glycogenolysis in mouse cortical slices: a possible regulatory mechanism for the local control of energy metabolism. Proc Natl Acad Sci U S A.

[30] Vu JP, Larauche M, Flores M, Luong L, Norris J, Oh S (2015). Regulation of appetite, body composition, and metabolic hormones by vasoactive intestinal polypeptide (VIP). J Mol Neurosci.

[31] Arden GB, Barnard WM, Mushin AS (1974). Visually evoked responses in amblyopia. Br J Ophthalmol.

[32] Snyder A, Shapley R (1979). Deficits in the visual evoked potentials of cats as a result of visual deprivation. Exp Brain Res.

[33] Jang J, Kyung SE (2018). Assessing amblyopia treatment using multifocal visual evoked potentials. BMC Ophthalmol.

[34] Hubel DH, Wiesel TN (1970). The period of susceptibility to the physiological effects of unilateral eye closure in kittens. J Physiol.

[35] Duffy KR, Lingley AJ, Holman KD, Mitchell DE (2016). Susceptibility to monocular deprivation following immersion in darkness either late into or beyond the critical period. J Comp Neurol.

[36] Montey KL, Quinlan EM (2011). Recovery from chronic monocular deprivation following reactivation of thalamocortical plasticity by dark exposure. Nat Commun.

[37] Mitchell DE, MacNeill K, Crowder NA, Holman K, Duffy KR (2016). Recovery of visual functions in amblyopic animals following brief exposure to total darkness. J Physiol.

[38] Suzuki M, Nagae M, Nagata Y, Kumagai N, Inui K, Kakigi R (2015). Effects of refractive errors on visual evoked magnetic fields. BMC Ophthalmol.

[39] Gozes I, Bardea A, Reshef A, Zamostiano R, Zhukovsky S, Rubinraut S (1996). Neuroprotective strategy for Alzheimer disease: intranasal administration of a fatty neuropeptide. Proc Natl Acad Sci U S A.

[40] Dufes C, Olivier JC, Gaillard F, Gaillard A, Couet W, Muller JM (2003). Brain delivery of vasoactive intestinal peptide (VIP) following nasal administration to rats. Int J Pharm.

[41] Duffy KR, Fong MF, Mitchell DE, Bear MF (2018). Recovery from the anatomical effects of long-term monocular deprivation in cat lateral geniculate nucleus. J Comp Neurol.

